# International Society of Sports Nutrition position stand: meal frequency

**DOI:** 10.1186/1550-2783-8-4

**Published:** 2011-03-16

**Authors:** Paul M La Bounty, Bill I Campbell, Jacob Wilson, Elfego Galvan, John Berardi, Susan M Kleiner, Richard B Kreider, Jeffrey R Stout, Tim Ziegenfuss, Marie Spano, Abbie Smith, Jose Antonio

**Affiliations:** 1 Dept. of Health, Human Performance and Recreation, Baylor University, Waco, TX, USA; 2 School of Physical Education and Exercise Science, University of South Florida, Tampa, FL, USA; 3 Department of Exercise Science and Sports Studies, The University of Tampa, Tampa, FL, USA; 4 Dept. of Exercise and Nutrition Sciences, University at Buffalo, Buffalo, NY, USA; 5 Precision Nutrition Inc., Toronto, ON Canada; 6 High Performance Nutrition, Mercer Island, WA, USA; 7 Department of Health and Kinesiology, Texas A & M University, College Station, TX, USA; 8 The University of Oklahoma, Norman, OK, USA; 9 The Center for Applied Health Sciences, Stow, Ohio, USA; 10 Spano Sports Nutrition Consulting, Atlanta, GA, USA; 11 Department of Exercise Science and Biology, Nova Southeastern University, Fort Lauderdale, FL, USA

## Abstract

Position Statement: Admittedly, research to date examining the physiological effects of meal frequency in humans is somewhat limited. More specifically, data that has specifically examined the impact of meal frequency on body composition, training adaptations, and performance in physically active individuals and athletes is scant. Until more research is available in the physically active and athletic populations, definitive conclusions cannot be made. However, within the confines of the current scientific literature, we assert that:

1. Increasing meal frequency does not appear to favorably change body composition in sedentary populations.

2. If protein levels are adequate, increasing meal frequency during periods of hypoenergetic dieting may preserve lean body mass in athletic populations.

3. Increased meal frequency appears to have a positive effect on various blood markers of health, particularly LDL cholesterol, total cholesterol, and insulin.

4. Increased meal frequency does not appear to significantly enhance diet induced thermogenesis, total energy expenditure or resting metabolic rate.

5. Increasing meal frequency appears to help decrease hunger and improve appetite control.

The following literature review has been prepared by the authors in support of the aforementioned position statement.

## Introduction

Among adults 20 years or older, living in the United States, 65.1% are classified as overweight or obese [1]. Furthermore, there is no indication that this trend is improving [1]. Excess body fat has potential physical and psychological health implications as well as potential negative influences on sport performance as well. The various dietary aspects that are associated with overeating and obesity are not well understood [2]. One debated area that is often purported to play a role in body weight/composition changes is meal frequency. The amount and type of calories consumed, along with the frequency of eating, is greatly affected by sociological and cultural factors [3]. Recent evidence suggests that the frequency in which one eats may also be, at least in part, genetically influenced [4]. Infants have a natural desire to eat small meals (i.e., nibble) throughout the day [5]. However, as soon as a child reaches a certain age he/she is trained to consume meals in a generally predictable manner [5]. In the modernized world, meal frequency is affected by cultural/social norms as well as an individual's personal beliefs about his/her health or body composition. According to a study utilizing data from the 1987-1988 Nationwide Food Consumption Survey (NFCS), the average daily meal frequency for the 3,182 American adults that completed the study was 3.47 [6]. If meals that consisted of less than or equal to 70 kcals, (primarily consisting of tea, coffee, or diet beverages) were excluded from the analysis, the number decreased to 3.12 meals per day. These habits closely mirror the traditional three meals per day pattern (i.e., breakfast, lunch, and dinner) that is common throughout the industrialized world. Although it is often suggested that "nibblers" or "grazers" (i.e., defined in much of the pertinent literature as those that eat smaller meals, but more frequently throughout the day) may be at a metabolic advantage as compared to the "gorgers" (i.e., those that eat fewer, but larger meals), the evidence is inconclusive. Some scientists have theorized that consuming a small number of larger meals throughout the day may lead to increased obesity possibly due to increased fat synthesis and storage (i.e., lipogenesis) following a meal [7]. However, there remains debate within the scientific community as the available data is still somewhat equivocal.

In the last few years, studies on the effects of meal frequency have been encouraged among researchers [8]. A majority of this research is justifiably centered on the obesity epidemic. Unfortunately, there is very limited data that has examined the impact of meal frequency on body composition, training adaptations, and performance in physically active individuals and athletes. The primary purpose of this position stand is to discuss the various research findings in which meal/eating frequency has been an independent variable in human studies that assess body composition, various health markers, thermic effect of food (a.k.a. diet induced thermogenesis), energy expenditure, nitrogen retention, and satiety. Also, an attempt has been made to highlight those investigations that have included athletes and physically active individuals in interventions that varied meal frequency eating patterns.

## Body Weight and Body Composition

### Observational Studies

Several studies utilizing animal models have demonstrated that meal frequency can affect body composition [9-12]. Specifically, an inverse relationship between meal frequency and body composition has been reported [9-12]. Some of the earliest studies exploring the relationship between body weight and meal frequency in humans were published approximately 50 years ago. Table 1 and 2 provide a brief summary of several observational (i.e., cross-sectional, prospective, etc.) human studies that have examined the effect of meal frequency on body weight and/or body composition.

**Table 1:** Observational Studies Supporting the Effectiveness of Increased Meal Frequency on Weight loss/Fat loss

Study (year)	Population	Measurements	Findings
Fabry et al.[13] (1964)	379 older males (60-64 yrs)	Frequency of food intake survey, calculation to determine overweight classification, triceps and subscapular skinfolds, and blood variables	Ingesting > 5meals/d, as compared to < 3 meals/d, significantly improves overweight classification and subcutaneous fat.
Hedja & Fabry [14] (1964)	89 males (30-50 yrs)	2 week diet records along with height, body weight, and 12 site skinfold thickness	The group that ate less than 4 meals/day had a significantly greater body mass and skinfold averages than those that ate > 5 meals/day.
Metzner et al. [15] (1977)	948 males and 1,080 females (35-69 yrs)	24 hour diet record interview, calculated adiposity index (i.e., calculated using triceps and subscapular skinfold measurements, height, and weight)	Adiposity index was inversely related (significantly) to meal frequency in both men and women after adjusting for caloric intake. In summary, as meal frequency increased, overweight classification decreased.
Drummond et al. [16] (1998)	42 males and 37 females (20-55 yrs) with a BMI from 18-30. (Suspected under-reporters were excluded from final analysis)	7 day food diary; 7 day activity diary, 48 hour HR monitoring, 4 site skinfold thickness, height, and body weight.	Significant negative correlation between eating frequency and body weight was observed in males, but not females. Eating frequency was significantly correlated with total energy intake in females, but not in males. In both men and women no significant correlations between eating frequency and total energy expenditure were observed.
Ruidavets et al. [17] (2002)	330 males (45-64 yrs)	3 day diet record, estimated physical activity (i.e., leisure, work related, and walking/cycling to work), body mass index, and waist-to-hip ratio	After eliminating under reporters (new sample size = 297) and restrained eaters (new sample size = 243), a significant negative correlation between eating frequency and BMI as well as waist-to-hip ratio was observed.
Ma et al. [18] (2003)	251 males and 248 females (20-70 yrs)	24 hour dietary recalls, physical activity recalls, body weight, BMI, and physical activity recalls were collected every 3 months for 1 year	After adjusting for age, sex, physical activity, education, and total energy intake, participants reporting 4 or more eating episodes per day had a significantly lower risk of developing obesity than those eating 3 or fewer times per day.
Franko et al. [19] (2008)	1,209 black and 1,166 white female school children (9-19 yrs)	Multiple 3-day food diaries taken over several years, height, weight, and self reported physical activity	Girls between 9-19 years old, that ate 3 or more meals per day had significantly lower BMI-for-age Z scores.

**Table 2:** Observational Studies Refuting the Effectiveness of Increased Meal Frequency on Weight loss/Fat loss

Study (year)	Population	Measurements	Findings
Dreon et al. [20] (1988)	155 sedentary, overweight males (i.e., 120-140% of ideal weight) (30-59 yrs)	7 day diet records, physical activity questionnaires, VO2 max treadmill test, resting metabolic rate via indirect calorimetry, hydrostatic weighing, and body mass.	Meal frequency did not have a significant effect on percent body fat, total weight, fat-free mass, or resting metabolic rate.
Kant et al. [21] (1995)	2,580 males and 4,567 females (25-74 yrs)	Baseline 24-hour dietary recall that assessed meal frequency and compared to follow-up interview several years later. Body weight, BMI, and physical activity were also assessed.	When regression analysis accounted for various covariates (i.e., age, energy intake, level of physical activity, smoking status, race, education, baseline BMI, alcohol intake, and level of morbidity), no significant differences between weight change and meal frequency were reported either at baseline or the follow-up.
Summerbell et al. [22] (1996)	187 males and females (divided into 4 different age groups (adolescent, working age, middle aged, and elderly). Suspected under-reporters were excluded from final analysis	7 day dietary records and BMI	After removing suspected under-reporters from the analysis, only the adolescent group demonstrated a significant inverse relationship between meal frequency and BMI.
Anderson & Rossner [23] 1996)	86 obese and 61 normal weight males (20-60 yrs)	Multiple 24 hour dietary recalls (12 total) and BMI	No significant differences in food intake patterns were observed after suspected under-reporters were excluded from final analysis (obese: n = 23; normal weight: n = 44).
Crawley & Summerbell [24] (1997)	298 males and 433 females (16-17 yrs)	4 day dietary record and BMI	Initial analysis in both males and females revealed that there was a significant inverse relationship between feeding frequency and BMI. Removing suspected under-reporters still yielded a significant inverse relationship. However, after removing overweight male dieters and under-weight/normal weight females who believed they were overweight, no significant relationship between meal frequency and BMI was observed.
Titan et al. [25] (2001)	6,890 males and 7,776 females (45-75 yrs)	Food frequency questionnaire, BMI, waist-hip ratio (WHR), and self-reported occupational physical activity	After adjusting for confounding variables (i.e., smoking status, age, occupational activity, etc), no consistent significant association in males and females was observed when comparing individuals who ate 1-2 as compared to greater than 6 times per day to BMI or WHR.
Bertéus Forslund et al. [26] (2002)	83 obese and 94 normal weight reference women (37-60 yrs)	Meal pattern questionnaire and BMI	The obese women consumed a significantly greater 6.1 meals/day as opposed to the reference group (non-overweight women) which consumed 5.2 meals/day.
Pearcey and de Castro [27] (2002)	7 male and 12 female "weight gaining" college students and 7 males and 12 female "weight stable" matched controls (no age range reported)	7 day food intake diary, 7 day physical activity diary, and BMI	The observed weight gain in the "weight gaining" adults was attributed to the significantly greater intake of fat, carbohydrate, and overall food per meal, but not meal frequency.
Yannakoulia et al.[28] (2007)	64 pre and 50 post-menopausal women (including normal weight, overweight, and obese) (24-74 yrs) (Suspected under-reporters were excluded from analysis)	3 day food records, activity records, self-reported physical activity assessment, BMI, WHR, and body composition (dual x-ray absorptiometery)	There was no association between adiposity indices and eating frequency in pre-menopausal women, but there was a significant positive correlation between body fat percentage and meal frequency in post-menopausal women. Eating frequency was positively correlated with energy intake in both groups of women.
Howarth et al. [2] (2007)	1,792 younger (20-59 yrs) and 893 older (60-69 yrs) males and females (Suspected under-reporters were excluded from analysis)	Two 24 hour diet records and BMI	After adjusting for sex, age, smoking status, ethnicity, income, etc in both age groups, eating frequency was positively associated with energy intake. Older and younger individuals who ate more than three and six times a day, respectively, had a significantly higher BMI (i.e., in the overweight category) than those who ate less than three and six, respectively.
Duval et al. [29] (2008)	69 non-obese (BMI b/w 20-29 kg/m2), premenopausal women (48-55 yrs) (Suspected under-reporters were excluded from analysis)	7 day food diaries, body composition (dual x-ray absorptiometry), peak VO2, resting energy expenditure (REE) via indirect calorimetry, and physical activity energy expenditure (PAEE) using an accelerometer	A significant positive correlation was observed between eating frequency and total energy intake. There was an initial significant negative correlation between eating frequency and each of the following: BMI, body fat percentage and fat mass. However, after adjusting for PAEE and peak oxygen consumption, the associations were no longer significant.

The observational studies listed in Table 1 tend to support [13-19], while investigations in Table 2 refute [2,20-29] the effectiveness of increased meal frequency on body weight and/or body composition. Some of the aforementioned studies [13-15,18,19], if taken at face value, seem to effectively suggest a compelling negative correlation between meal frequency and body composition/body weight. However, aside from obvious genetic differences between subjects, there are other potential confounding factors that could alter the interpretation of these data. Studies in humans that have compared self-reported dietary intake to measured and/or estimated total daily energy expenditure have shown that under-reporting of food is not uncommon in both obese and non-obese individuals [30]. Several investigations have demonstrated that the under-reporting may be significantly greater in overweight and obese individuals [24,30-35]. Additionally, older individuals have also been shown to underreport dietary intake [36]. Under-reporting of dietary intake may be a potential source of error in some of the previously mentioned studies [13-15,18,19] that reported positive effects of increased meal frequency. In fact, in their well written critical review of the meal frequency research from ~1964-1997, Bellisle et al. [37] point this out and suggest that classification of subjects' meal frequency and under-reporting of dietary intake can potentially complicate the interpretation of these previously mentioned studies as well as future studies that explore this relationship. Bellisle and colleagues [37] also bring up the valid point of "reverse causality" in which someone who gains weight might skip meal(s) with the hope that they will lose weight. If an individual chooses to do this during the course of a longitudinal study, where meal frequency data is collected, it could potentially alter data interpretation to make it artificially appear that decreased meal frequency actually caused the weight gain [37]. However, even taking reverse causality into account, certain studies listed in Table 1 still demonstrated a positive effect of increased meal frequency on body weight/composition even after accounting for possible under-reporters [16,17] and dieters/restrained eaters [17]. Thus, the potential problem of under-reporting cannot be generalized to all studies that have shown a benefit of increased meal frequency.

Equally important, several studies that initially found a significant inverse relationship between meal frequency and body weight/composition were no longer significant after the investigators adjusted for under-reporters [22,23], dieters/restrained eaters [24], physical activity/peak oxygen consumption [29], or other various potential confounding variables such as age, energy intake, physical activity, smoking status, etc. [21]. Nevertheless, Ruidavets et al. [17] still demonstrated a significant negative correlation between meal frequency and both BMI and waist-to-hip ratio even after adjusting for under-reporters, and dieters.

Taking all of the observational studies listed in Table 1 and 2 into account, it is difficult to make definitive conclusions about the relationship between meal/eating frequency and body weight/composition. However, when accounting for the effects of under-reporting, exercise, and other confounding variables, the preponderance of the research suggests that increased meal frequency does not play a significant role in decreasing body weight/weight composition.

### Experimental Studies

The majority of experimental studies utilizing meal frequency interventions recruited overweight/obese populations [38-42]. When total daily calories were held constant (but hypocaloric) it was reported that the amount of body weight lost was not different even as meal frequency increased from a range of one meal per day up to nine meals per day [38-42]. Most recently in 2010, Cameron et al. [43] examined the effects of an eight week hypocaloric diet in both obese male and female participants. The subjects consumed either three meals per day (low meal frequency) or three meals plus three additional snacks (high meal frequency). Individuals in both the high and low meal frequency groups had the same caloric restriction (~700 kcals/day). Both groups lost ~5% of their initial weight as well as similar decreases in lean mass, fat mass and overall BMI [43]. There were no significant differences between the varying meal frequencies groups in any measure of adiposity [43].

In addition to overweight/obese populations, a few experimental investigations have been conducted in normal weight subjects [44-47]. In relation to improvements in body weight and body composition, the results were similar to those of the overweight/obese trials - no improvements with increasing meal frequencies [44-47]. Even under isocaloric conditions or when caloric intake was designed to maintain the subjects' current body weight, increasing meal frequency from one meal to five meals [47] or one meal to three meals [45] did not improve weight loss. One exception to the non-effectiveness of increasing meal frequency in bodyweight/composition was conducted by Fabry and coworkers [48]. The investigators demonstrated that increases in skinfold thickness were significantly greater when ingesting three meals per day as compared to five or seven meals per day in ~10-16 year old boys and girls. Conversely, no significant differences were observed in ~6-11 year old boys or girls [48].

Application to Nutritional Practices of Athletes: Based on the data from experimental investigations utilizing obese and normal weight participants, it would appear that increasing meal frequency would not benefit the athlete in terms of improving body composition. Interestingly, when improvements in body composition are reported as a result of increasing meal frequency, the population studied was an athletic cohort [49-51]. Thus, based on this limited information, one might speculate that an increased meal frequency in athletic populations may improve body composition. The results of these studies and their implications will be discussed later in the section entitled "Athletic Populations".

## Blood Markers of Health

Reduced caloric intake, in a variety of insects, worms, rats, and fish, has been shown to have a positive impact on health and lifespan [52-54]. Similarly, reduced caloric intake has been shown to have health promoting benefits in both obese and normal-weight adults as well [55]. Some of the observed health benefits in apparently healthy humans include a reduction in the following parameters: blood pressure, C-reactive protein (CRP), fasting plasma glucose and insulin, total cholesterol, LDL cholesterol, and atherosclerotic plaque formation [55]. However, much less has been published in the scientific literature regarding the effects of varying meal frequencies on markers of health such as serum lipids, serum glucose, blood pressure, hormone levels, and cholesterol.

Gwinup and colleagues [56,57] performed some of the initial descriptive investigations examining the effects of "nibbling" versus "gorging" on serum lipids and glucose in humans. In one study [57], five hospitalized adult women and men were instructed to ingest an isocaloric amount of food for 14 days in crossover design in the following manner:

• One large meal per day

• 10 meals per day given every two hours

• Three meals per day

"Gorging" (i.e., one meal per day) led to increases in serum lipids when compared to eating three meals per day. Conversely, 14 days of "nibbling" (i.e., 10 meals per day) led to small decreases in serum lipids such as serum phospholipids, esterified fatty acids, and cholesterol [57]. It is important to point out that this study only descriptively examined changes within the individual and no statistical analyses were made between or amongst the participants [57]. Other studies using obese [58] and non-obese [59] subjects also reported significant improvements in total cholesterol when an isocaloric amount of food was ingested in eight meals vs. one meal [58] and 17 snacks vs. 3 normal meals [59]. In a cross-sectional study which included 6,890 men and 7,776 women between the ages of 45-75 years, it was reported that the mean concentrations of both total cholesterol and LDL cholesterol significantly decreased with increased meal frequency in the general population, even after adjusting for possible confounding variables such as obesity, age, physical activity, and dietary intake [25]. Specifically, after adjusting for confounding variables, the mean total and LDL cholesterol concentrations were ~5% lower in the individuals that ate more than six times a day as opposed to those only eating once or twice per day [25]. Similarly, Edelstein and colleagues [60] reported that in 2,034 men and women aged 50-89, the individuals that ate greater than or equal to four times per day had significantly lower total cholesterol than those who ate only one to two meals per day. Equally important, LDL concentrations were also lower in those who ate with greater frequency [60].

A more recent study examined the influence of meal frequency on a variety of health markers in humans [45]. Stote et al. [45] compared the effects of consuming either three traditional meals (i.e., breakfast, lunch, and dinner) or one large meal on markers of health. The study was a randomized, crossover study in which each participant was subjected to both meal frequency interventions for eight weeks with an 11 week washout period between interventions [45]. All of the study participants ingested an amount of calories needed to maintain body weight, regardless if they consumed the calories in either one or three meals per day. The individuals who consumed only one meal per day had significant increases in blood pressure, and both total and LDL cholesterol [45].

In addition to improvements with lipoproteins, there is evidence that increasing meal frequency also exerts a positive effect on glucose kinetics. Gwinup et al., [5,56] along with others [13], have reported that "nibbling" or increased meal frequency improved glucose tolerance. Specifically, when participants were administered 4 smaller meals, administered in 40 minute intervals, as opposed to one large meal of equal energy density, lower glucose and insulin secretion were observed [61]. Jenkins and colleagues [59] demonstrated no significant changes in serum glucose concentrations between diets consisting of 17 snacks compared to three isocaloric meals per day. However, those that ate 17 snacks per day significantly decreased their serum insulin levels by 27.9% [59]. Ma et al. [18] point out that the decrease in serum insulin with increased meal frequency may decrease body fat deposition by decreasing lipase enzyme activity.

Contrary to the aforementioned studies, some investigations using healthy men [62], healthy women [63], and overweight women [39] have reported no benefits in relation to cholesterol and triglycerides. Although not all research agrees regarding blood markers of health such as total cholesterol, LDL cholesterol, and glucose tolerance, it appears that increasing meal frequency may have a beneficial effect. Mann [64] concluded in his review article that there seems to be no deleterious effects in regard to plasma lipids or lipoproteins by eating a relatively large number of smaller meals. It is noted, however, that the studies where benefits have been observed with increased meal frequency have been relatively short and it is not known whether these positive adaptations would occur in longer duration studies [64].

Application to Nutritional Practices of Athletes: Although athletic and physically active populations have not been independently studied in this domain, given the beneficial outcomes that increasing meal frequency exerts on a variety of health markers in non-athletic populations, it appears as if increasing meal frequency in athletic populations is warranted in terms of improving blood markers of health.

## Metabolism

Metabolism encompasses the totality of chemical reactions within a living organism. In an attempt to examine this broad subject in a categorized manner, the following sections will discuss the effects of meal frequency on:

• Diet induced thermogenesis (i.e., DIT or also known as the thermic effect of food)

• Resting metabolic rate/total energy expenditure

• Protein Metabolism

### Diet Induced Thermogenesis

It is often theorized that increased eating frequency may be able to positively influence the thermic effect of food, often referred to as diet induced thermogenesis (DIT), throughout the day as compared to larger, but less frequent feedings [65]. Kinabo and Durnin [65] investigated this theory when they instructed eighteen non-obese females to consume either a high carbohydrate-low fat diet consisting of 70%, 19%, and 11% or a low carbohydrate-high fat diet consisting of 24%, 65% and 11% from carbohydrate, fat and protein, respectively [65]. Each diet was isocaloric and consisted of 1,200 kcals. In addition, on two different instances, each participant consumed their meal either in one large meal or as two smaller meals of equal size. The investigators observed no significant difference in the thermic effect of food either between meal frequencies or between the compositions of the food [65].

In two other studies utilizing normal-weight young women [66] and obese children [67] as subjects, it was reported that the ingestion of one large meal significantly increased resting energy expenditure/thermic effect of food as compared to an isocaloric food intake that was ingested in either six [66] or three [67] smaller meals. LeBlanc et al. [61] tested the thermic effect of food in six individuals after consuming four small meals as opposed to one large meal of equal caloric density. Contrary to the earlier findings of Tai et al. [66], post-prandial thermogenesis and fat utilization was greater in the group that consumed the smaller, more frequent meals [61].

Smeets and colleagues [68] conducted a very practical study comparing the differences in consuming either two or three meals a day in normal weight females in energy balance. In this randomized, crossover design in which participants consumed the same amount of calories over a traditional three meal pattern (i.e., breakfast, lunch, and dinner) compared to just two meals (breakfast and dinner) it was demonstrated that there was no significant difference on diet induced thermogenesis when measured over 36 hours in a respiration chamber [68]. However, by consuming three meals per day, fat oxidation, measured over 24 hours using deuterium labeled fatty acids was significantly greater and carbohydrate oxidation was significantly lower when compared to eating just two meals per day [68].

### Resting Metabolic Rate/Total Energy Expenditure

It is argued that the best methodology to study the effects of meal frequency on metabolism utilizes a metabolic/respiratory chamber (i.e., a whole body calorimeter). While these conditions are not free living, these types of studies are able to control extraneous variables to a greater extent than other methods. Four investigations utilizing overweight/obese participants [40,41,69,70] and one investigation examining normal-weight participants [7] confined the participants to either a metabolic/respiration chamber [7,41,69,70] or a confined metabolic unit [40] and reported that there were no improvements in resting metabolic rate or 24-hour energy expenditure due to increasing the number of meals ingested. In each of these investigations, the same number of calories were ingested over the duration of a day, but the number of meals ingested to consume those calories varied from one vs. three and five feedings [40], two vs. three to five feedings [41], two vs. seven feedings [7,70], and two vs. six feedings [69]. The amount of time the participants were confined to the metabolic/respiratory chambers or metabolic unit ranged from a few hours [7] to a few days [41,69,70] to several weeks [40]. From the aforementioned studies examining the effect of meal frequency on the thermic effect of food and total energy expenditure, it appears that increasing meal frequency does not statistically elevate metabolic rate.

### Protein Metabolism

Garrow et al., [40] reported that during a hypocaloric diet lasting three weeks in obese subjects, nitrogen loss was significantly less when the diet consisted of 15% protein as opposed to 10% protein. Additionally, nitrogen loss was also significantly less when five versus one meal per day were consumed and protein was kept at a constant 13% [40]. Equally important, the lowest nitrogen loss occurred when five versus one meal per day were consumed and protein content was 15% versus 10% [40]. The authors concluded that the protein content of total caloric intake is more important than the frequency of the meals in terms of preserving lean tissue and that higher protein meals are protein sparing even when consuming low energy intakes [40]. While this study was conducted in obese individuals, it may have practical implications in athletic populations. Specifically, the findings support the idea that frequent feedings with a higher protein content (15% vs. 10%) may reduce nitrogen losses during periods of hypocaloric intake.

In contrast to the Garrow et al. findings, Irwin et al. [63] compared the effects of different meal composition and frequency on nitrogen retention. In this study, healthy, young women consumed either three meals of equal size, three meals of unequal size (two small and one large), or six meals (calorie intake was equal between groups). The investigators reported that there was no significant difference in nitrogen retention between any of the different meal frequency regimens [63].

Finkelstein and Fryer [39] also reported no significant difference in nitrogen retention, measured through urinary nitrogen excretion, in young women who consumed an isocaloric diet ingested over three or six meals. The study lasted 60 days, in which the participants first consumed 1,700 kcals for 30 days and then consumed 1,400 kcals for the remaining 30 days [39]. The protein and fat content during the first 30 days was 115 and 50 grams, respectively, and during the last 30 days 106 grams of protein and 40 grams of fat was ingested. The protein content was relatively high (i.e., ~27% - 30% of the total daily calories) and may have aided in the nitrogen retention that was observed. Similarly, in a 14-week intervention, Young et al., [42] reported that consuming 1,800 kcals fed as one, three, or six meals a day did not have a significant impact on nitrogen retention in 11 moderately obese, college aged men.

It is important to emphasize that the previous studies were based on the nitrogen balance technique. Nitrogen balance is a measure of whole body protein flux, and may not be an ideal measure of skeletal muscle protein metabolism. Thus, studies concerned with skeletal muscle should analyze direct measures of skeletal muscle protein synthesis and breakdown (i.e., net protein synthesis). Based on recent research, it appears that skeletal muscle protein synthesis on a per meal basis may be optimized at approximately 20 to 30 grams of high quality protein, or 10-15 grams of essential amino acids [71-73]. In order to optimize skeletal muscle protein balance, an individual will likely need to maximize the response on a per meal basis. Research shows that a typical American diet distributes their protein intake unequally, such that the least amount of protein is consumed with breakfast (~10-14 grams), while the majority of protein is consumed with dinner (~29-42 grams) [74]. Thus, in the American diet, protein synthesis would likely only be optimized once per day with dinner. This was recently demonstrated by Wilson et al. [75] in a published abstract (utilizing a rodent model). The investigators found that equally distributing protein over three meals (16% per meal) resulted in greater overall protein synthesis and muscle mass, in comparison to providing suboptimal protein (8%) at breakfast and lunch, and greater than optimal protein (27%) with dinner [75]. In eucaloric meal frequency studies, which spread protein intake from a few (i.e., two to three meals) to several meals (i.e., greater than five meals), the bolus of protein per meal shrinks, which may provide several suboptimal, or possibly non-significant rises in protein synthesis as opposed to a few meals which may maximally stimulate protein synthesis. This is likely the case in the previously mentioned study by Irwin et al [63] who compared three ~20 gram protein containing meals, to six ~10 gram protein containing meals. Such a study design may negate any positive effects meal distribution could have on protein balance.

With this said, in order to observe the true relationship between meal frequency and protein status, studies likely need to provide designs in which protein synthesis is maximized over five-six meals as opposed to three meals. This was demonstrated by Paddon-Jones and colleagues [76] who found that mixed muscle protein synthesis was ~23% greater when consuming three large ~850-calorie meals (~23 g protein, ~127 g carbohydrate, and ~30 g fat), supplemented with an additional three small 180-calorie meals containing 15 grams of essential amino acids, as compared to just three 850-calorie meals alone. In summary, the recent findings from the Wilson study [75] combined with the results published by Paddon-Jones et al. [76] suggest that when protein synthesis is optimized, increased feeding frequency may positively impact protein status.

The inattention paid to protein intake in previously published meal frequency investigations may force us to reevaluate their utility. Nutrient timing research [77,78] has demonstrated the importance of protein ingestion before, during, and following physical activity. Therefore, future research investigating the effects of meal frequency on body composition, health markers, and metabolism should seek to discover the impact that total protein intake has on these markers and not solely focus on total caloric intake.

Application to Nutritional Practices of Athletes: Athletic and physically active populations have not been independently studied in relation to increasing meal frequency and observing the changes in resting metabolic rate/total energy expenditure. Considering the data published in overweight/obese and normal weight populations, it appears as if increasing meal frequency would not improve resting metabolic rate/total energy expenditure in physically active or athletic populations. In regards to protein metabolism, it appears as if the protein content provided in each meal may be more important than the frequency of the meals ingested, particularly during hypoenergetic intakes.

## Hunger and Satiety

Research suggests that the quantity, volume, and the macronutrient composition of food may affect hunger and satiety [79-83]. However, the effect of meal frequency on hunger is less understood. Speechly and colleagues [83] examined the effect of varying meal frequencies on hunger and subsequent food intake in seven obese men. The study participants consumed 1/3 of their daily energy requirement in one single pre-load meal or evenly divided over five meals administered hourly. The meals consisted of 70% carbohydrate, 15% protein, and 15% fat. Several hours after the initial pre-load meal(s), another meal (i.e., lunch) was given to the participants *ad libitum *to see if there was a difference in the amount that was consumed following the initial pre-load meal(s). The scientists reported that when the single pre-load meal was given, participants consumed 27% (i.e., ~358 kcals) more energy in the *ad libitum *meal than those who ate the multiple pre-load meals [83]. Interestingly, this difference occurred even though there were no significant changes in subjective hunger ratings [83]. Another study with a similar design by Speechly and Buffenstein [84] demonstrated greater appetite control with increased meal frequency in lean individuals. The investigators also suggest that eating more frequent meals might not only affect insulin levels, but may affect gastric stretch and gastric hormones that contribute to satiety [84].

Stote et al. [45] reported that there were significantly greater increases in hunger in individuals eating only one meal as compared to three meals per day. In addition, Smeets and colleagues [68] demonstrated that consuming the same energy content spread over three (i.e., breakfast, lunch, and dinner) instead of two (i.e., breakfast and dinner) meals per day led to significantly greater feeling of satiety over 24 hours [68]. To the contrary, however, Cameron and coworkers [43] reported that there were no significant differences in feelings of hunger or fullness between individuals that consumed an energy restricted diet consisting of either three meals per day or three meals and three snacks. Furthermore, the investigators also determined that there were no significant differences between the groups for either total ghrelin or neuropeptide YY [43]. Both of the measured gut peptides, ghrelin and neuropeptide YY, are believed to stimulate appetite.

Although all research does not agree, it appears that the preponderance of the available research suggests that eating more frequently may decrease hunger and/or food intake at subsequent meals. Even if nothing else was directly affected by varying meal frequency other than hunger alone, this could possibly justify the need to increase meal frequency if the overall goal is to suppress the feeling of hunger.

Application to Nutritional Practices of Athletes: Athletic and physically active populations have not been independently studied in relation to increasing meal frequency and observing the changes in subjective hunger feelings or satiety. Utilizing data from non-athletic populations, increasing meal frequency would likely decrease feelings of hunger and/or food intake at subsequent meals for athletes as well. For athletes wishing to gain weight, a planned nutrition strategy should be implemented to ensure hyper-energetic eating patterns.

## Athletic Populations

To date, there is a very limited research that examines the relationship of meal frequency on body composition, hunger, nitrogen retention, and other related issues in athletes. However, in many sports, including those with weight restrictions (gymnastics, wrestling, mixed martial arts, and boxing), small changes in body composition and lean muscle retention can have a significant impact upon performance. Therefore, more research in this area is warranted.

In relation to optimizing body composition, the most important variables are energy intake and energy expenditure. In most of the investigations discussed in this position stand in terms of meal frequency, energy intake and energy expenditure were evaluated in 24-hour time blocks. However, when only observing 24-hour time blocks in relation to total energy intake and energy expenditure, periods of energy imbalance that occurs within a day cannot be evaluated. Researchers from Georgia State University developed a method for simultaneously estimating energy intake and energy expenditure in one-hour units (which allows for an hourly comparison of energy balance) [50]. While this procedure is not fully validated, research has examined the relationship between energy deficits and energy surpluses and body composition in elite female athletes. In a study by Duetz et al. [50], four groups of athletes were studied: artistic and rhythmic gymnasts (anaerobic athletes), and middle-distance and long-distance runners (aerobic athletes). While this study did not directly report meal frequency, energy imbalances (energy deficits and energy surpluses), which are primarily influenced through food intake at multiple times throughout the day were assessed. When analyzing the data from all of the elite female athletes together, it was reported that there was an approximate 800 kilocalorie deficit over the 24-hour data collection period [50]. However, the main purpose of this investigation was to determine energy imbalance not as a daily total, but as 24 individual hourly energy balance estimates. It was reported that the average number of hours in which the within-day energy deficits were greater than 300 kcal was about 7.5 hours, while the average number of hours where the within-day energy surpluses were greater than 300 kcal was about three hours (which makes sense since these athletes were consuming a hypocaloric diet) [50]. When data from all the athletes were combined, energy deficits were positively correlated with body fat percentage, whereas energy surpluses were negatively correlated with body fat percentage. Similarly, the total hours with deficit kcals was positively correlated with body fat percentage, while the total hours with surplus kcals were negatively correlated with body fat percentage. It is also interesting to note that an energy surplus was (non-significantly) inversely associated with body fat percentage. In light of these findings, the authors concluded that athletes should not follow restrained or delayed eating patterns to achieve a desired body composition [50].

Iwao and colleagues [51] examined boxers who were subjected to a hypocaloric diet while either consuming two or six meals per day. The study lasted for two weeks and the participants consumed 1,200 kcals per day. At the conclusion of the study, overall weight loss was not significantly different between the groups [51]. However, individuals that consumed 6 meals per day had significantly less loss of lean body mass and urinary 3-methylhistidine/creatinine as opposed to those that only consumed two meals [51]. This would suggest that an increased meal frequency under hypocaloric conditions may have an anti-catabolic effect.

A published abstract by Benardot et al. [49] demonstrated that when a 250 calorie snack was given to 60 male and female college athletes for two weeks after breakfast, lunch, and dinner, as opposed to a non-caloric placebo, a significant amount of fat (-1.03%) was lost and lean body mass (+1.2 kg) gained. Furthermore, a significant increase in anaerobic power and energy output was observed via a 30-second Wingate test in those that consumed the 250 calorie snack [49]. Conversely, no significant changes were observed in those consuming the non-caloric placebo. Interestingly, when individuals consumed the total snacks of 750 kcals a day, they only had a non-significant increase in total daily caloric consumption of 128 kcals [49]. In other words, they concomitantly ate fewer calories at each meal. Lastly, when the 250 kcal snacks were removed, the aforementioned values moved back to baseline levels 4 weeks later [49].

In conclusion, the small body of studies that utilized athletes as study participants demonstrated that increased meal frequency had the following benefits:

• suppression of lean body mass losses during a hypocaloric diet [51]

• significant increases in lean body mass and anaerobic power [49] (abstract)

• significant increases in fat loss [49] (abstract)

These trends indicate that if meal frequency improves body composition, it is likely to occur in an athletic population as opposed to a sedentary population. While no experimental studies have investigated why athletes may benefit more from increased meal frequency as compared to sedentary individuals, it may be due to the anabolic stimulus of exercise training and how ingested nutrients are partitioned throughout the body. It is also possible that a greater energy flux (intake and expenditure) leads to increased futile cycling, and over time, this has beneficial effects on body composition.

Even though the relationship between energy intake and frequency of eating has not been systematically studied in athletes, available data demonstrates that athletes (runners, swimmers, triathletes) follow a high meal frequency (ranging from 5 to 10 eating occasions) in their daily eating practices [85-88]. Such eating practices enable athletes to ingest a culturally normalized eating pattern (breakfast, lunch, and dinner), but also enable them to adhere to the principles of nutrient timing (i.e., ingesting carbohydrate and protein nutrients in the time periods before and immediately following physical activity/competition).

## Conclusion

Like many areas of nutritional science, there is no universal consensus regarding the effects of meal frequency on body composition, body weight, markers of health, markers of metabolism, nitrogen retention, or satiety. The equivocal outcomes of the studies that have examined the relationship between meal frequency and body composition may be attributed to under-reporting of food intake (especially in overweight or obese individuals), the various ages of participants, and whether or not exercise/physical activity was accounted for in the analysis. Furthermore, it has been pointed out by Ruidavets et al. [17] that the various ways a meal versus a snack is defined may lead to a different classification of study participants and ultimately influence the outcome of a study. Equally important, calculating actual meal frequency, especially in free-living studies, depends on the time between meals, referred to as "time lag", and may also influence study findings [17]. Social and cultural definitions of an actual "meal" (vs. snack) vary greatly and time between "meals" is arbitrary [17]. In other words, if the "time-lag" is very short, it may increase the number of feedings as opposed to a study with a greater "time-lag" [17]. Thus, all of these potential variables must be considered when attempting to establish an overall opinion on the effects of meal frequency on body composition, markers of health, various aspect of metabolism, and satiety. Taking all of this into account, it appears from the existing (albeit limited) body of research that increased meal frequency may not play a significant role in weight loss/gain when under-reporting, restrained eating, and exercise are accounted for in the statistical analyses. Furthermore, most, but not all of the existing research, fails to support the effectiveness of increased meal frequency on the thermic effect of food, resting metabolic rate, and total energy expenditure. However, when energy intake is limited, increased meal frequency may likely decrease hunger, decrease nitrogen loss, improve lipid oxidation, and improve blood markers such as total and LDL cholesterol, and insulin. Nonetheless, more well-designed research studies involving various meal frequencies, particularly in physically active/athletic populations are warranted.

## Competing interests

The authors declare that they have no competing interests.

## Authors' contributions

All authors read and extensively reviewed and contributed to the final manuscript.
